# Engineered EVs with pathogen proteins: promising vaccine alternatives to LNP-mRNA vaccines

**DOI:** 10.1186/s12929-024-01000-1

**Published:** 2024-01-17

**Authors:** Bin Zhang, Wei Kian Sim, Tang-Long Shen, Sai Kiang Lim

**Affiliations:** 1 Institute of Molecular and Cellular Biology, A*STAR, 8A Biomedical Grove, Singapore, 138648 Singapore; 2 Paracrine Therapeutics Pte. Ltd., 10 Choa Chu Kang Grove #13-22 Sol Acres, Singapore, 688207 Singapore; 3 Department of Plant Pathology and Microbiology, National Taiwan University, Taipei, 10617 Taiwan; 4 Center for Biotehnology, National Taiwan University, Taipei, 10617 Taiwan; 5 Department of Surgery, YLL School of Medicine, National University of Singapore (NUS), Lower Kent Ridge Road, Singapore, 119074 Singapore

**Keywords:** EV-based protein vaccines, Extracellular vesicles (EVs), Exosomes, LNP-mRNA vaccines, Mesenchymal stem/stromal cell (MSC)

## Abstract

Extracellular vesicles (EVs) are tiny, lipid membrane-bound structures that are released by most cells. They play a vital role in facilitating intercellular communication by delivering bioactive cargoes to recipient cells and triggering cellular as well as biological responses. EVs have enormous potential for therapeutic applications as native or engineered exosomes. Native EVs are naturally released by cells without undergoing any modifications to either the exosomes or the cells that secrete them. In contrast, engineered EVs have been deliberately modified post-secretion or through genetic engineering of the secreting cells to alter their composition. Here we propose that engineered EVs displaying pathogen proteins could serve as promising alternatives to lipid nanoparticle (LNP)-mRNA vaccines. By leveraging their unique characteristics, these engineered EVs have the potential to overcome certain limitations associated with LNP-mRNA vaccines.

## Background

Extracellular vesicles (EVs) are small lipid membrane-bound vesicles that are released by almost all types of cells. They play important roles in intercellular communication and contain a diverse range of biomolecules, including proteins, nucleic acids, and lipids [1]. Two better known types of EVs are exosomes and microvesicles, which differ in terms of size, composition, and biogenesis mechanisms. Exosomes are generally smaller in size (30–150 nanometers) and originate from the endosomal compartment of the cell. They are formed through the inward budding of multivesicular bodies (MVBs) and are released into the extracellular environment upon fusion of MVBs with the plasma membrane. Microvesicles, on the other hand, are larger (100–1000 nanometers) and are generated through the direct outward budding and shedding of the plasma membrane. Although various EV types have been described, a universal marker that definitively identifies a specific EV type is currently lacking. Researchers rely on a combination of methods such as size-based isolation, specific protein markers, and electron microscopy to characterize and classify EV populations. It is worth noting that ongoing research efforts are focused on identifying more precise markers and developing improved techniques for EV isolation and analysis. For a more comprehensive understanding of EVs, readers should refer to the MISEV2018 guidelines [2], which provide detailed information on EV isolation, characterization, and functional studies. These guidelines were established by the International Society for Extracellular Vesicles (ISEV) and serve as a valuable resource for researchers in the field.

EVs have emerged as promising tools in various fields, including therapeutics, diagnostics, and drug delivery [3]. They have been reported to be therapeutically efficacious in many animal models of disease and some EVs have already entered clinical trials. For example, 45 clinical trials on MSC-EVs are currently registered on https://clinicaltrials.gov/ (searched on 25 April 2023 for (“extracellular vesicles” OR “exosomes”) Mesenchymal|Interventional Studies). They are also thought to have unique characteristics such as a capacity to carry bioactive molecules and inherent targeting abilities that make them attractive candidates for targeted drug delivery systems [4]. Additionally, EVs hold great potential as diagnostic biomarkers for various diseases, as their cargo reflects the physiological and pathological state of their parent cells. Moreover, EVs can be harnessed for therapeutic purposes, as they can deliver therapeutic cargo to target cells and modulate biological processes.

One of the earliest applications of EVs [1] is in the development of vaccines, particularly in cancer immunotherapeutic vaccines. While the term “exosomes” was widely used in early studies with an assumption of endosomal biogenesis, there was no definitive evidence that the EVs being studied were exosomes. Nonetheless, several seminal observations in the 1980 and 1990 s initiated the development of EV-based cancer immunotherapeutic vaccines.

In 1986, Schirrmacher and Barz [5] reported that tumor cells secrete tumor antigens in exosomes and these exosomes exert anti-tumor effects on cytotoxic T lymphocytes (CTLs). Altieri et al. [6] subsequently demonstrated that exosomes prepared from plasmacytoma cells in vitro were prophylactic in protecting mice from challenged with plasmacytoma. In 1996, Raposo et al. [7] demonstrated that exosomes derived from both human and murine B lymphocytes induced antigen-specific MHC class II-restricted T cell responses. In 1998, Zitvogel et al. reported that exosomes derived from tumor peptide-pulsed dendritic cells (DCs) express tumor antigens with functional MHC class I and II molecules, enabling to induce in vivo CTL priming and consequent tumor growth suppression [8]. They further demonstrated that when tumor-derived exosomes from different tumors were loaded onto DCs, they triggered T cell–mediated anti-tumor immune responses leading to rejections of autologous tumors and strong inter-tumor cross-protections.

Consequently, DC-derived exosomes (Dexs) pulsed with tumor peptides or tumor-derived exosomes were tested as a cancer vaccine candidate in three phase 1 clinical trials. A retrospective review in 2016 of these clinical trials by Zitvogel and colleagues opined that while Dex is generally safe and feasible, Dex-stimulated T cell responses displayed weak in contrast to pre-clinical studies [9]. Unexpectedly, Dexs were found to stimulate NK cells. The review concluded that more work is required to develop Dex as a cancer vaccine and overcome the lack of T cell response.

Similar research has also been conducted on EV-based prophylactic vaccines against infectious diseases. This research was triggered by observations that cells infected with viruses, parasites, or bacteria produce EVs that carry pathogen-associated antigens, which have the potential to activate immune cells. For example, studies have shown that macrophages infected with *Mycobacterium tuberculosis*, *M bovis BCG*, *Salmonella typhimurium*, *Toxoplasma gondii*, and *Mycobacterium avium* secrete EVs that carry bacterial and parasitic antigens, and these EVs can elicit an immune response [10, 11]. It was later reported that exosomes from macrophages infected with *M. tuberculosis* or pulsed with *M. tuberculosis* proteins can protect mice from *Mycobacterium tuberculosis* infection [12]. However, there have been no clinical trials testing these vaccines, and their clinical efficacy remains unknown.

EVs have also been implicated in various stages of viral infection, where they can either enhance or inhibit viral infections (reviewed by [13]). While some evidence suggests that EVs carry viral antigens, such as observed in serum EVs from pigs infected with African Swine Fever Virus [14], it is unclear whether EVs from virally infected cells could be used as vaccines. Early studies using EVs from murine Lymphocytic Choriomeningitis Virus (LCMV)-infected DCs reported that these EVs did not confer vaccine protection against acute LCMV infection [15], similar to the observations with Dex. Despite the presence of viral antigens in these EVs, it remains uncertain whether they can provide effective vaccine protection against the respective viruses.

In summary, EV-based vaccines have undergone clinical testing mainly in cancer and these are summarized in Table 1. All the trials consistently reported the safety of EV-based vaccines, though their overall efficacy was generally limited. While these initial clinical studies have provided valuable insights into the role of EVs in stimulating immune responses and carrying pathogen-associated antigens, their efficacy as prophylactic vaccines has been underwhelming. One reason for this lackluster performance is that the first generation of EV vaccines were made by exposing cells to antigens or cells infected with pathogens without considering their immunogenicity or stability. Notably, there have been no recent clinical trials for EV-based vaccines. The majority of recent EV clinical trials focused on utilizing EVs as vehicles for therapeutic delivery or as therapeutic agents.

**Table 1:** Clinical trials using exosomes as a potential vaccine

Name	Sponsor	Start time	Status	Conditions	NCT ID	Results	Refs.
Dex (DC-derived exosomes pulsed with MAGE peptides)	Institut Gustave Roussy	2001 (Phase 1)	Completed	Metastatic melanoma	N.A.	Safe, well-tolerated, induction of NK cell activity	[16]
	Duke University Medical Center; Anosys Inc.	2002 (Phase 1)	Completed	Advanced Non-small cell lung cancer (NSCLC)	N.A.	Safe, well-tolerated, induction of NK cell activity	[17]
Dex2 (DC-derived exosomes pulsed with IFN-γ)	Gustave Roussy, Cancer Campus, Grand Paris	2010 (Phase 2)	Completed	Advanced NSCLC after chemotherapy	NCT01159288	No cancer-specific T-cell response, induction of NKp30 activation	[18]
Aexs (ascites-derived exosomes) plus GM-CSF	The Fourth Hospital Affiliated to Guangxi Medical University	2006 (Phase 1)	Completed	Advanced colorectal cancer	N.A.	Safe, well-tolerated, strong anti-tumor CTL response	[19]
iExosomes (MSC-derived exosomes with KRAS G12D siRNA)	M.D. Anderson Cancer Center	2021 (Phase 1)	Ongoing	Metastatic pancreas cancer with KrasG12D mutation	NCT03608631	N.A.	N.A.
CF-CB-MSCs-EVs (cell free cord blood MSC-derived extracellular vesicles)	General Committee of Teaching Hospitals and Institutes, Egypt	2014 (Phase 2/3)	Completed	Chronic kidney diseases (type 1 diabetes, interstitial nephritis)	N.A.	Improved overall kidney function, safety and tolerance	N.A.
		2014 (Phase 2/3)	Unknown status	Type 1 diabetes mellitus	NCT02138331	N.A.	N.A.
MSC-Exos (adipose MSC-derived exosomes)	Ruijin Hospital	2020 (Phase 1)	Completed	Healthy	NCT04313647	Safety and tolerance of aerosol inhalation	[20]
MSC-Exos (cord blood MSC-derived exosomes)	Tianjin Medical University	2017 (Phase 1)	Unknown status	Refractory macular holes	NCT03437759	N.A.	N.A.
EXO-CD24 (Exosomes overexpressing CD24)	Tel-Aviv Sourasky Medical Center	2020 (Phase 1)	Unknown status	Moderate or severe COVID-19 infection	NCT04747574	N.A.	N.A.
	Athens Medical Society	2021 (Phase 2)	Unknown status	Moderate or severe COVID-19 infection	NCT04902183	N.A.	N.A.
MSC-derived exosomes (aerosol inhalation of adipose MSC-derived exosomes)	Ruijin Hospital	2020 (Phase 1)	Completed	Severe Novel Coronavirus Pneumonia	NCT04276987	Safe, well-tolerated	[21]
EXO inhalation (aerosol inhalation of bone marrow MSC-derived exosomes)	Olga Tyumina	2020 (Phase 2)	Unknown status	Covid-19, SARS-CoV-2 pneumonia	NCT04602442	N.A.	N.A.
Intravenous injection of MSC-derived exosomes	AVEM HealthCare	2023 (Phase 1/2)	Not yet recruiting	Covid19, Novel Coronavirus Pneumonia, Acute Respiratory Distress Syndrome	NCT04798716	N.A.	N.A.
EXO inhalation (aerosol inhalation of MSC-derived exosomes)	State-Financed Health Facility “Samara Regional Medical Center Dinasty”	2020 (Phase 1/2)	Completed	Covid19, SARS-CoV-2 Pneumonia	NCT04491240	Safe and efficient	N.A.
CSTC-Exo (COVID-19 Specific T Cell derived exosomes)	TC Erciyes University	2020 (Phase 1)	Unknown status	Corona Virus Infection, Pneumonia	NCT04389385	N.A.	N.A.

## Next-Generation EV vaccines

Recent advancements in EV technology have opened up new possibilities for the rational design of EVs to present highly effective antigens in their native-like conformations on the EV membrane. One notable example is the work by Choi’s group, who has made significant progress by integrating a reversible protein-protein interaction module controlled by blue light into the exosome biogenesis process [22]. This technology has allowed for the successful loading of specific proteins into the lumen or membrane of exosomes. Additionally, Codiak has developed a high-density EV engineering platform that involves tethering exogenous payload proteins to proteins that are preferentially sorted into EVs as luminal or membrane proteins [23]. These technologies pave the way for the creation of designer EVs capable of presenting the most effective antigens for vaccination.

Utilizing proteins rather than mRNA to load EVs for vaccine development offers several advantages. Protein-based vaccines have a well-established history of triggering safe and robust immune responses, with reliable production, storage, transportation, and distribution infrastructure in place. This existing infrastructure allows for efficient manufacturing and global accessibility of protein-based vaccines. In contrast, mRNA vaccines require stringent ultra-cold storage and transportation, which poses challenges to their universal distribution and accessibility.

Although lipid nanoparticles (LNPs) could also be loaded with proteins, the choice of proteins are limited to purified synthetic or soluble recombinant proteins [24]. They cannot accommodate transmembrane glycoproteins in their native-like conformations which are essential for effective display of antigenic epitopes [25]. In general, membrane-bound antigens such as those in the membranes of cell/EV are considered more accessible for recognition by B cells and other APCs [26–28].

The immunogenicity of EV membrane-bound proteins has been reported to be superior to cell membrane-bound proteins [28]. When intramuscularly injected, EV membrane-bound GFP enhances both humoral and cell-mediated responses to a greater extent than its secreted, intracellular, or cell membrane-bound counterparts in mice. These responses encompass both IgA and IgG, indicative of a robust and diverse immune reaction that include both mucosal and systemic immunity. Taken together, these findings also strongly suggest that mRNA vaccines, which generate protein antigens within cells for secretion or display on the cell membrane, may not be as effective in eliciting an immune response as protein antigens exhibited on EV membranes.

Therefore, EV-protein vaccine holds great potential as a platform for the development of vaccines, including cancer immunotherapeutic vaccines and prophylactic vaccines against infectious diseases. While the initial studies have laid the foundation for understanding the role of EVs in immune stimulation and antigen delivery, further research is necessary to optimize EV vaccines by engineering them to carry specific proteins and enhance their immunogenicity. With ongoing advancements in EV engineering and understanding of their biological functions, EVs present immense potential as a versatile platform for the advancement of vaccines, encompassing cancer immunotherapeutic vaccines and prophylactic vaccines targeting infectious diseases.

In this discussion, we will explore the numerous advantages offered by EVs as a delivery platform for protein vaccines in comparison to LNP-mRNA vaccines.

## Circumventing limitations of LNP-mRNA COVID-19 vaccines with EV-based protein vaccines?

LNP-mRNA vaccines have emerged as the primary and highly effective approach for immunization against SARS-CoV-2 and possibly other infectious diseases as well. Nonetheless, despite their success, this technology does have intrinsic limitations that have posed challenges to its overall effectiveness. In this context, EV-protein vaccines offer a promising solution to overcome these limitations and enhance the field of vaccination.

### LNP toxicity

One critical limitation of LNP-mRNA vaccines is the inherent toxicity of the LNPs used to deliver mRNA vaccine [29, 30]. In fact, the adverse side effects observed in LNP-mRNA vaccines, such as the Pfizer/BioNTech and Moderna vaccines, have been attributed to the inflammatory response induced by LNPs [31]. As such there are intense research studies to overcome this toxicity and improve safety and efficacy of LNPs for RNA-based therapeutics. For example, some of these efforts involve the synthesis of novel lipid chemical structures and the incorporation of different types of helper lipids and lipopolymers into nanoparticle formulations [32]. However, it remains to be seen if the toxicity of LNPs can be effectively improved.

**Fig. 1:**
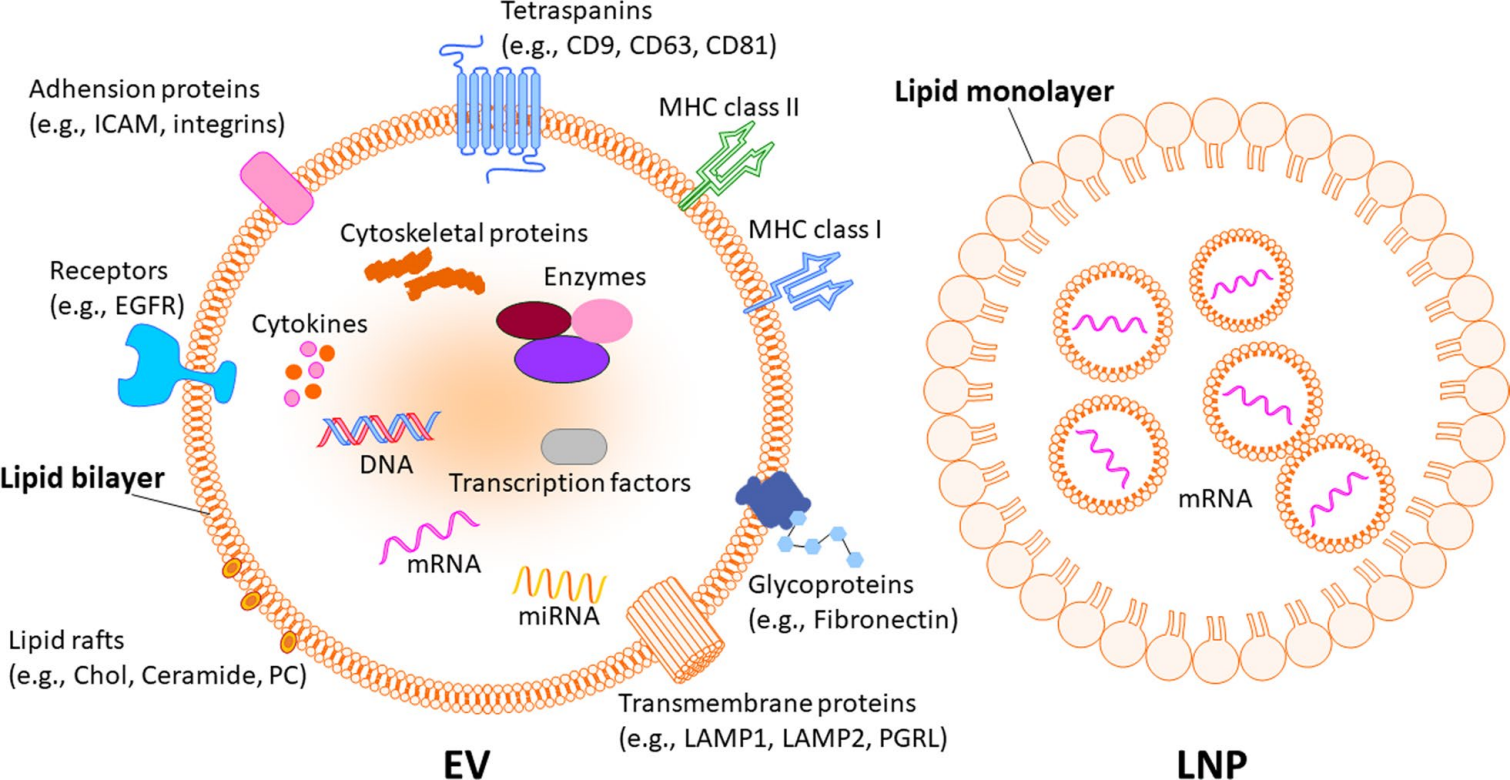
The general structure of an EV and LNP. EV possesses a membrane structure made of a cell homologous lipid bilayer and carry a variety of biologically active substances such as proteins, nucleic acids, glycoproteins, metabolites. Some proteins represent EV markers (e.g., tetraspanins CD9, CD63, CD81), while other proteins are variable depending on the cell type origin, including adhesion molecules (ICAM and integrins), major histocompatibility (MHC) molecules, enzymes, and other factors. LNP is currently recognized as promising candidates for transferring vaccine mRNA, owing to their stability and biocompatibility. Typically, LNPs consist of four key components: cationic lipids, ionizable lipids, polyethylene glycols (PEGs), and cholesterol. The LNPs could be either mono- or bi-layer. These components collaborate synergistically to enhance the effective delivery of mRNA into the cytoplasm. *EV* extracellular vesicle, *LNP* lipid nanoparticle

In contrast, EVs offer inherent safety advantages compared to LNPs. Unlike LNPs, which have been documented in scientific literature to have toxic properties, EVs have not been reported to exhibit significant toxicity. This can be attributed to the fact that EVs are natural components present in all bodily fluids. A depiction of the general structure of EVs and LNPs is illustrated in Fig. 1. The safety of EV-rich tissues, such as blood and serum, has been extensively investigated in routine medical procedures like transfusion, establishing a solid foundation for the overall safety of EV treatments. Furthermore, clinical trials examining the use of exosomes or EVs for interventional studies further support their safety profile. Of the 21 clinical trials conducted as of 12th April 2023 (source: https://clinicaltrials.gov/), four have already been completed without any adverse events, emphasizing the favorable safety record of EVs as therapeutics or delivery vehicles.

In summary, the inherent toxicity associated with LNPs poses a critical limitation in their use as delivery vehicles, necessitating ongoing intensive research to enhance their safety and efficacy. In contrast, EVs exhibit remarkable safety advantages over LNPs, supported by the absence of documented toxicity and extensive research on the safety of EV-rich tissues. These characteristics make EVs highly promising for vaccine development, especially in scenarios requiring long-term or repeated use.

### Scalable manufacture

Scalable manufacture of mRNA vaccines presents challenges due to its non-continuous manufacturing process. Instead of a single streamlined process, it involves multiple discrete steps, including the production and purification of linearized DNA templates, transcription of the DNA to generate mRNA, capping of the mRNA, purification of the mRNA, encapsulation of the mRNA in LNPs, and finally fill and finish [33]. Moreover, the materials required for each of these steps are expensive and limited in supply, which further increases the cost and complexity of scaling up production [33].

On the other hand, the production of EV-protein vaccines can be achieved using two primary strategies. One approach involves obtaining EVs from cells genetically modified to express protein vaccines in EVs, while the other involves engineering isolated EVs to express protein vaccines. The former approach typically employs a large-scale, continuous manufacturing process, which begins with cell expansion, followed by the harvesting of the conditioned medium, EV enrichment, and fill and finish [34]. The latter method requires an additional step of engineering the native EVs produced by unmodified cells. Generally, manufacturing EV vaccines utilizing genetically modified cells offers clear advantages over the alternative strategy.

It is worth noting that many parallels can be drawn between the well-established manufacturing of cells for cell therapy or biologics, such as MSCs or antibodies, and the production of EVs from cells. For instance, the manufacturing process for MSC-EVs shares many similarities with that of MSCs themselves [35]. Since EVs are non-living entities, there is no need to implement the expensive monitoring and mitigation processes required for MSC products to ensure a viable living state. This factor can offset any additional manufacturing costs associated with MSC-EV production.

Independent of the processes used in the manufacturing of either EV- or LNP-based vaccines will have to adhere strictly to regulatory guidelines (http://www.fda.gov/downloads/BiologicsBloodVaccines/GuidanceComplianceRegulatoryInformation/Guidances/Vaccines/ucm092272.pdf). Specifically, the manufacturing processes, product characteristics, and product testing which are collectively known as CMC must be defined in order to to ensure that vaccines are safe, effective and consistent between batches [36]. The Table 2 provides an overview of the differences in key CMC of EV- vs. LNP-based vaccines.

**Table 2:** Differences in CMC considerations for EVs vs. LNPs as vaccine vehicles

Aspect	EV-based vaccines	LNP-based vaccines
Product Characteristics	Complex Characteristics requiring multiple assays e.g., NTA, TEM, mass spectrometry, ELISA, flow cytometry	Precise lipid composition: standardized lipid, size, and charge characterization
Manufacturing Process	A biological process using eukaryotic cells	A chemical process where the RNA is synthesized by enzymatic reaction or by microbials
Product testing	Cell debris, protein aggregates purity	Lipid and chemical/biological reactants; Elimination of contaminants through purification steps; Meticulous control of lipid purity and reactants

Overall, although the manufacturing of EV-protein vaccines utilizing living cells might appear intimidating, it is not inherently more challenging than the production of LNP-mRNA vaccines. The scalability and continuous manufacturing precedents established in the field of cell therapy and biologics can be easily applied to EV and EV vaccine production. This has the potential to make EV production as, if not more cost competitive as the discontinuous manufacturing of mRNA vaccines.

### Instability of product

Unlike most traditional vaccines that can be stored at 2–8 °C (https://ldh.la.gov/assets/oph/Center-PHCH/Center-PH/immunizations/vaccine-storage-handling.pdf), the current LNP-mRNA COVID-19 vaccines developed by Moderna and BioNTech/Pfizer require ultra-cold temperatures for storage. Moderna’s vaccine needs to be kept between − 15 and − 25 °C(https://www.cdc.gov/vaccines/covid-19/info-by-product/moderna/downloads/storage-summary.pdf), while BioNTech/Pfizer’s vaccine requires storage temperatures between − 60 and − 90 °C (https://www.cdc.gov/vaccines/covid-19/info-by-product/pfizer/downloads/storage-summary.pdf). This presents significant challenges for distribution due to limited infrastructure for ultra-low temperature storage and transportation.

The reasons for the differing storage requirements of Moderna and BioNTech/Pfizer COVID-19 vaccines are not fully understood. Although significant improvements have been achieved in enhancing the stability and efficacy of mRNA-LNP vaccines, a notable gap still exists in our understanding of their long-term storage stability. To address this issue, it is imperative to adopt a systematic approach that enables the identification and understanding of the physicochemical degradation mechanisms affecting LNP-mRNA stability [37, 38]. This will help in the design of stable LNP-mRNA formulations that can be stored, transported and administered at refrigerated or ambient temperatures.

Recent studies have shown promising results regarding the stability of mRNA-LNP formulations. Some formulations have demonstrated the ability to be lyophilized and stored at room temperature or even up to 37 °C for several weeks [39, 40]. However, further validation is required to ensure reproducibility and reliability of these findings.

In the case of EVs, which are also generally stored at − 20 °C, lyophilization has become a common practice. Lyophilized EVs have been reported to remain stable at higher temperatures, such as 25 °C for four weeks [41] and 40 °C for three weeks [42]. Lyophilized EVs have also been utilized for bio-functional testing in pre-clinical animal models [43, 44].

Although both LNP-mRNAs and EVs are typically unstable and require ultra-low temperature storage, there is increasing evidence that they can be lyophilized and stored at higher temperatures in their lyophilized states. This advancement has the potential to make LNP-mRNA or EV-protein vaccines more accessible by eliminating the need for ultra-cold storage and transportation.

### Circumventing poor endosomal escape

**Fig. 2:**
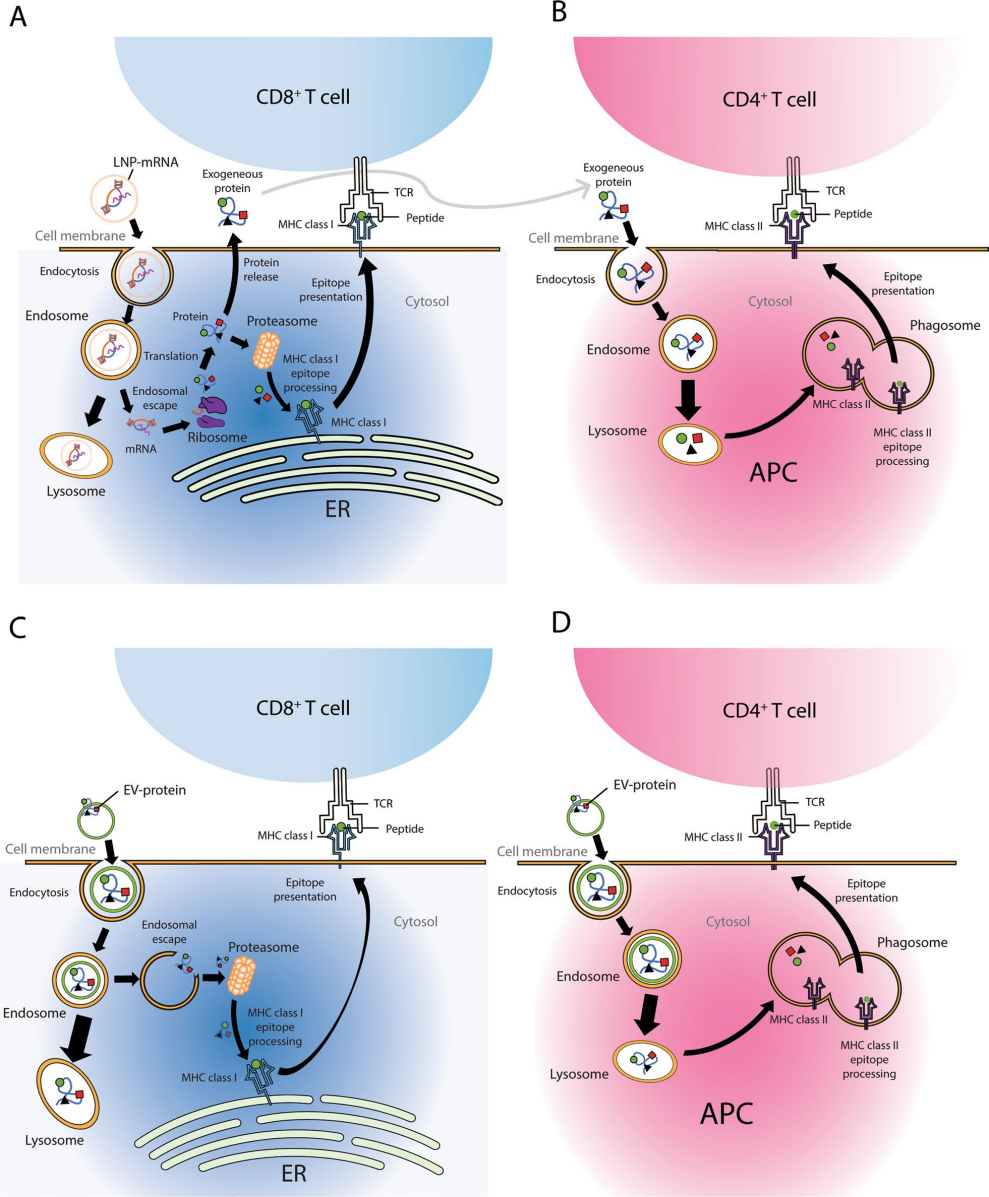
Endocytosis of LNP-mRNAs / EV-proteins for immune interaction. **A** Most of the internalized LNP-mRNAs will be shuttled to the lysosome and a small fraction will escape from the endosomes to release mRNA for protein translation. Some of the newly translated proteins could undergo degradation by the proteasome for MHC class I antigen presentation to CD8^+^ T cells Others could be displayed on the cell surface or secreted into the extracellular space. **B** The membrane-bound or secreted proteins when endocytosed by APCs will be processed in the lysosomes for MHC class II antigen presentation to CD4^+^ T cells. **C** Most of the internalized EV-proteins will be shuttled to the lysosome and a small fraction will escape from the endosomes to release protein for degradation by the proteasome and MHC class I antigen presentation to CD8^+^ T cells. **D** When EV-proteins are endocytosed by APCs, the poor endosomal escape enhances lysosomal processing of the proteins and facilitates MHC class II antigen presentation to CD4^+^ T cells. *ER* endoplasmic reticulum, *TCR* T cell receptor, *APC *antigen-presenting cell

Both EVs and LNPs face a critical challenge as delivery vehicles for vaccines, which is their limited ability to escape the endosome-lysosome pathway after being internalized by cells. Recent research suggests that only a small fraction of LNPs (approximately 1–2%) can successfully escape from the endosomes and avoid degradation in the lysosomes [45, 46]. The principal challenge in RNA drug development lies in achieving efficient cytosolic delivery, hindered by the inefficiency of traversing both plasma and endosomal membranes to reach the cytosol [47]. In the context of LNP-mRNAs, once the LNPs successfully escape, they liberate their mRNA cargo within the cytoplasm, facilitating the translation of RNA into proteins. These proteins can then undergo proteolysis via the ubiquitin proteasome pathway to facilitate antigen presentation on MHC-class I, which is necessary for generating an immune response (Fig. 2A). Alternatively, the proteins may either be displayed on the cell surface membrane or released into the extracellular space [48, 49], where they can be endocytosed by antigen-presenting cells (APCs) for presentation on MHC-class II [48, 49] (Fig. 2B).

Although EVs also exhibit inefficient endosomal escape when internalized by cells [50, 51], this is less detrimental in terms of immune system recognition of EV-protein vaccines. EV-protein vaccines, unlike LNP-mRNA vaccines, do not require translation once they have escaped from the endosomes. Instead, they can undergo immediate proteolysis by the ubiquitin-proteasome system and subsequent loading onto MHC-class I for antigen presentation [48, 49]. (Fig. 2C). They also do not require translation to be presented on the cell membrane or secreted into the extracellular space for recognition by APCs. Furthermore, the poor endosomal escape of endocytosed EV-protein vaccines in APCs would likely promote lysosomal processing of the protein vaccines, facilitating antigen presentation on MHC-class II [48, 49] (Fig. 2D). Therefore, EV-based protein vaccines possess a significant advantage over LNP-mRNA vaccines in terms of endosomal escape and subsequent antigen presentation.

Another advantage of EVs as protein vaccine delivery systems over LNP-mRNA vaccines is the potential for wider dissemination of the protein from the site of administration to immune inductive sites [28]. In contrast, the protein antigen produced by the translation of the mRNA vaccine is retained at the cellular site of mRNA translation thereby reducing the accessibility of the translated proteins to immune cells.

Overall, EV-based protein vaccines are promising alternatives to LNP-mRNA vaccines, particularly in terms of endosomal escape and antigen presentation. In LNP-mRNA vaccines, the inability to escape from the endosome can result in the degradation of the vaccine in the lysosomes, thereby limiting its efficacy. In contrast, in EV-protein vaccines, even if they fail to escape the endosome in APCs, the proteins can still be processed in the lysosomes and presented by APCs to activate the immune system. This makes EV-based protein vaccines a more reliable and efficient option for inducing an immune response compared to LNP-mRNA vaccines.

### Overcoming weak mucosal immunity

The COVID-19 pandemic has significantly accelerated the progress of vaccine development, evaluation, manufacturing, and deployment across various platforms. Notably, mRNA-based vaccines, viral-vectored vaccines, inactivated virus-based vaccines, and subunit recombinant proteins have emerged as prominent approaches. According to a comprehensive review by Mouro and Fischer [52], over 10.5 billion doses of COVID-19 vaccines have been administered worldwide in just over a year, highlighting the scale of the vaccination efforts. By March 2022, ten vaccines had received emergency or full use approval from WHO-recognized regulatory authorities, with additional authorizations granted in specific countries. Furthermore, there are currently 346 COVID-19 vaccine candidates in development, with 151 undergoing clinical trials.

The review underscores the efficacy of most vaccines in preventing symptomatic infection, reducing the severity of illness, and decreasing mortality rates in both clinical trials and real-world settings. However, it also acknowledges that these vaccines have not proven effective in suppressing community transmission of the virus. Consequently, non-pharmaceutical protective measures like mask wearing, social distancing, and border closures have remained necessary. Unfortunately, these measures have imposed a significant socioeconomic cost, disproportionately impacting vulnerable populations.

Retrospective analyses have indicated that the current generation of LNP-mRNA COVID-19 vaccines has primarily been unsuccessful in suppressing community transmission due to their limited ability to induce mucosal immunity, despite their success in stimulating systemic immunity [52–55]. Since viruses such as SARS-CoV-2, avian influenza, SARS, MERS, and Nipah primarily target the upper respiratory tract and establish colonization on mucosal surfaces, effective prevention of such viral infections requires measures that impede initial mucosal colonization. In this regard, bolstering mucosal frontline immunity becomes crucial as it plays a critical role in preventing mucosal colonization by these viruses. By effectively impeding mucosal colonization, the subsequent dissemination of the virus into systemic tissues can be curtailed. This notion finds support in a recent report demonstrating that mice, even after receiving two intramuscular applications of an adenovirus-based mRNA vaccine, still require additional intranasal boosts with the same vaccine to stimulate high levels of mucosal IgA and lung-resident memory T cells and achieved complete protection against SARS-CoV-2 infection [56]. However, intranasal immunization with these adenoviral vectors did not elicit the expected pre-clinical response in a human clinical trial [57].

The current LNP-mRNA COVID-19 vaccines are administered intramuscularly, and they induce robust systemic immunity but weak mucosal immunity. To date, there have been no reports on intranasal administrations of LNP-mRNA vaccines to induce a mucosal immune response. In contrast to LNP-mRNA vaccines, intramuscular injection of EV membrane bound GFP enhances both IgA and IgG production, indicating that intramuscular injection of EV-protein vaccines can stimulate both mucosal and systemic immune responses [28]. Moreover, EV membrane decorated with a recombinant SARS-CoV-2 receptor-binding domain can be administered through inhalation and provide protection against live SARS-CoV-2 challenge in animals [44].

These findings underscore the significance of mucosal immunity in preventing mucosal infection and reducing the risk of severe systemic diseases. Vaccines based on EV-protein, which are more amenable to mucosal vaccination, such as intranasal administration, may hold promise for inducing effective mucosal immunity.

### Response to new emerging pathogens and variants

One of the advantages of LNP-mRNA vaccines is the rapid response time in developing a vaccine. In principle, the mRNA can be rapidly designed in silico and then chemically or biochemically synthesized to produce the LNP-mRNA as soon as the pathogen genome is known as well as the viral antigen candidate is identified. By the same token, the DNA sequence of viral antigen candidates can be readily inserted into EV engineering platforms as such those described above [22, 23] to generate EVs carrying the desired protein antigens.

## Conclusion

In conclusion, EVs offer a highly promising platform for the development of protein-based vaccines, presenting several advantages over LNP-mRNA vaccines. EVs possess unique characteristics, such as their ability to transport bioactive molecules and their natural targeting abilities, making them attractive candidates for targeted drug delivery systems and diagnostic biomarkers. Recent advancements in EV technology have facilitated the rational design of EVs, enabling the effective presentation of antigens in their native-like conformations on the EV membrane.

Compared to LNP-mRNA vaccines, EV-protein vaccines offer notable benefits. Firstly, EVs demonstrate inherent safety advantages as they have shown minimal toxicity, unlike LNPs. EVs are natural components found in all bodily fluids, and their safety has been extensively investigated in routine medical procedures. Secondly, the scalable production of EV-protein vaccines can be achieved using continuous manufacturing processes that are similar to well-established cell therapy or biologics production. This provides advantages over the non-continuous and complex manufacturing process of mRNA vaccines, which require expensive materials and face limited supply. Thirdly, mRNA vaccines require ultra-low temperature storage due to their instability, posing challenges for distribution. In contrast, EV-protein vaccines can potentially be stored at traditional refrigeration temperatures, simplifying storage and transportation logistics. In addition, EV-based protein vaccines could potentially mitigate the challenge of poor endosomal escape encountered by both EVs and LNPs, rendering them more reliable and efficient in stimulating an immune response. Furthermore, EVs exhibit a greater potential for widespread dissemination of the antigenic proteins compared to LNP-mRNA vaccines. Moreover, EV-protein vaccines have shown promise in inducing effective mucosal immunity, which is crucial for preventing viral infections. Lastly, development of EV-based vaccines has the potential for a rapid response time and flexibility that is on par with LNP-mRNA vaccines, enabling their adaptability to emerging pathogens and variants. While EV-based vaccines offer many advantages, they are not without their limitations. It is essential to consider these limitations when evaluating their suitability for specific applications. Some of the key limitations of EVs include: (1) EVs are a heterogeneous population of vesicles, and achieving consistency in EV preparations could be challenging; (2) EVs may lack the ability to precisely target specific cells or tissues, especially when compared to some synthetic delivery systems designed for precise targeting; (3) In the complex milieu of biological fluids, EVs could be subject to degradation and clearance, which may affect their efficacy; (4) EVs may face biological barriers, including the need to cross tissue barriers, reach specific cellular compartments, or escape lysosomal degradation, which could be influenced by factors such as tissue type and disease state. In Table 3, the advantages and disadvantages of EV-protein vaccines are delineated in comparison to LNP-mRNA vaccines.

**Table 3:** Comprehensive summary for the comparison of EV-protein vaccines and LNP-mRNA vaccines

Aspect	EV-Protein vaccines	LNP-mRNA vaccines
Advantages		
Immune response	Superior immunogenicity with both humoral and cell-mediated responses	Limited ability to induce mucosal immunity, primarily systemic
Storage and distribution	Efficient global manufacturing and accessibility	Ultra-cold storage requirements pose distribution challenges
Protein loading	Accommodates transmembrane glycoproteins for effective antigen display	Limited to purified synthetic or soluble recombinant proteins
Safety	Inherent safety, no documented significant toxicity	Inherent toxicity associated with LNPs, leading to adverse effects
Scalable mManufacturing	Large-scale, continuous manufacturing process	Non-continuous, complex, and expensive manufacturing process
Stability	Could be lyophilized. No need for cold chain logistics	Ultra-cold storage requirements. Required cold chain logistics
Antigen presentation	Direct antigen presentation	Required additional step of translation for presentation
Mucosal immunity	Potential to stimulate both mucosal and systemic responses	Primarily induces systemic immunity, weak in mucosal immunity
Rapid response to emerging pathogens	Yes	Yes
Disadvantages		
Heterogeneity	EVs are a heterogeneous population; achieving consistency may be challenging	LNPs are chemically constituted and are more precisely defined
Targeting precision	EV targeting is not well defined	LNPs designed for specific targeting may have higher targeting precision
Stability in biological fluids	EVs are subjected to the same degradation and clearance as most biologicals in biological fluids	LNPs as synthetics could be engineered to overcome biological degradation
Biological barriers	EVs may face challenges in crossing tissue barriers and escaping lysosomal degradation	LNPs may also encounter biological barriers, influencing efficacy

In conclusion, EV-protein vaccines have immense potential as a versatile prophylactic vaccine platform to provide both mucosal and systemic protection against infectious diseases.

## Data Availability

Not applicable.

## Abbreviations

CTLs: Cytotoxic T lymphocytes
DCs: Dendritic cells
Dexs: DC-derived exosomes
EVs: Extracellular vesicles
ISEV: International Society for Extracellular Vesicles
LCMV: Lymphocytic choriomeningitis virus
LNP: Lipid nanoparticle
MSC: Mesenchymal stem/stromal cell
MVBs: Multivesicular bodies
